# Metformin Suppresses Monocyte Inflammation and Metabolic Reprogramming by SARS-CoV-2 Spike Protein

**DOI:** 10.1093/geroni/igab046.1284

**Published:** 2021-12-17

**Authors:** Theodore Cory, Russell Emmons, Johnathan Yarbro, Brandt Pence

**Affiliations:** 1 University of Tennessee Health Science Center, Memphis, Tennessee, United States; 2 University of Memphis, Memphis, Tennessee, United States

## Abstract

COVID-19 disproportionately affects older adults, and a hallmark of the disease is a hyperinflammatory state that is associated with severity. Various anti-inflammatory therapeutics have shown mixed efficacy in treating COVID-19, and the mechanisms by which hyperinflammation occurs are not well understood. Previous research indicated that monocytes, a key innate immune cell, undergo metabolic reprogramming and produce inflammatory cytokines when stimulated with SARS-CoV-2. We hypothesized that binding by the viral spike protein mediates this effect, and that drugs which regulate immunometabolism – including the geroprotector drug metformin – could inhibit the inflammatory response in monocytes. Monocytes stimulated with recombinant SARS-CoV-2 spike protein subunit 1 showed a dose-dependent increase in glycolytic metabolism that was associated with production of pro-inflammatory cytokines including interleukin-6 and tumor necrosis factor-alpha. This response was dependent on hypoxia-inducible factor-1alpha, as chetomin inhibited glycolysis and cytokine production. Inhibition of glycolytic metabolism by 2-deoxyglucose (2-DG) or glucose deprivation also inhibited the glycolytic response, and 2-DG strongly suppressed cytokine production. Glucose-deprived monocytes rescued cytokine production by upregulating oxidative phosphorylation, an effect which was not present in 2-DG-treated monocytes due to the known effect of 2-DG on suppressing mitochondrial metabolism. Finally, pre-treatment of monocytes with metformin strongly suppressed spike protein-mediated cytokine production in monocytes, and abrogated glycolytic and mitochondrial metabolism. In summary, the SARS-CoV-2 spike protein induces a pro-inflammatory immunometabolic response in monocytes that can be suppressed by treatments that interfere with glycolytic activation, including metformin. This has potential implications for the treatment of hyperinflammation during COVID-19, which disproportionately affects older adults.

